# The Two-Device Problem: A Comprehensive Framework for Managing Transvalvular CIED Leads in the Era of Transcatheter Tricuspid Intervention

**DOI:** 10.3390/jcm15031303

**Published:** 2026-02-06

**Authors:** Mohammed Hussein Kamareddine, Edward M. Powers, Faisal Rahman, Ali R. Keramati, Konstantinos N. Aronis

**Affiliations:** 1 Division of Cardiology, Lankenau Medical Center, Main Line Health, Wynnewood, PA 19096, USA; husseinkamareddinem@mlhs.org; 2 Section of Cardiac Electrophysiology, Division of Cardiology, Johns Hopkins Hospital, Johns Hopkins University School of Medicine, Baltimore, MD 21287, USA; epower11@jh.edu; 3 Section of Intervention Cardiology, Division of Cardiology, Johns Hopkins Hospital, Johns Hopkins University School of Medicine, Baltimore, MD 21287, USA; frahman7@jhu.edu; 4 Section of Cardiac Electrophysiology of Electrophysiology, Division of Cardiology, Lankenau Medical Center, Main Line Health, Wynnewood, PA 19096, USA; keramatia@mlhs.org

**Keywords:** tricuspid regurgitation, transcatheter tricuspid valve intervention, cardiac implantable electronic device, transcatheter lead extraction, lead jailing, pacing strategies, implantable cardioverter-defibrillator, leadless pacemaker, Heart Team

## Abstract

Tricuspid regurgitation (TR) in patients with transvalvular cardiac implantable electronic device (CIED) leads is increasingly encountered as transcatheter tricuspid valve interventions (TTVI) expand, yet integrated guidance for managing this “two-device problem” remains limited. We performed a focused synthesis of contemporary evidence, organizing findings around mechanisms and diagnosis of TR in the setting of CIED leads, lead–device interactions across TTVI platforms, and clinical trade-offs of transvenous lead extraction (TLE) versus lead preservation or jailing. CIED-associated TR can arise from lead–leaflet impingement, leaflet injury, fibrotic adhesion, pacing-induced remodeling, or infection; true CIED-induced TR accounts for a minority of clinically significant TR, yet progression of TR after lead implantation occurs in 7–45% of patients, and moderate-to-severe TR in CIED populations is associated with 1.6- to 2.5-fold increased mortality risk. Lead conflict and lifetime consequences differ by TTVI modality: repair approaches are generally more lead-tolerant, whereas valve replacement creates obligate lead jailing with implications for lead performance, future extraction feasibility, and infection management. Management of TR with transvalvular CIED leads requires integrated Heart Team planning that anticipates downstream device needs. Standardized TR phenotyping, lead-aware TTVI selection, valve-sparing rhythm-device strategies, and structured post-procedural surveillance may improve outcomes; prospective studies are needed to define optimal extract-versus-jail pathways.

## 1. Introduction

**Figure 1:**
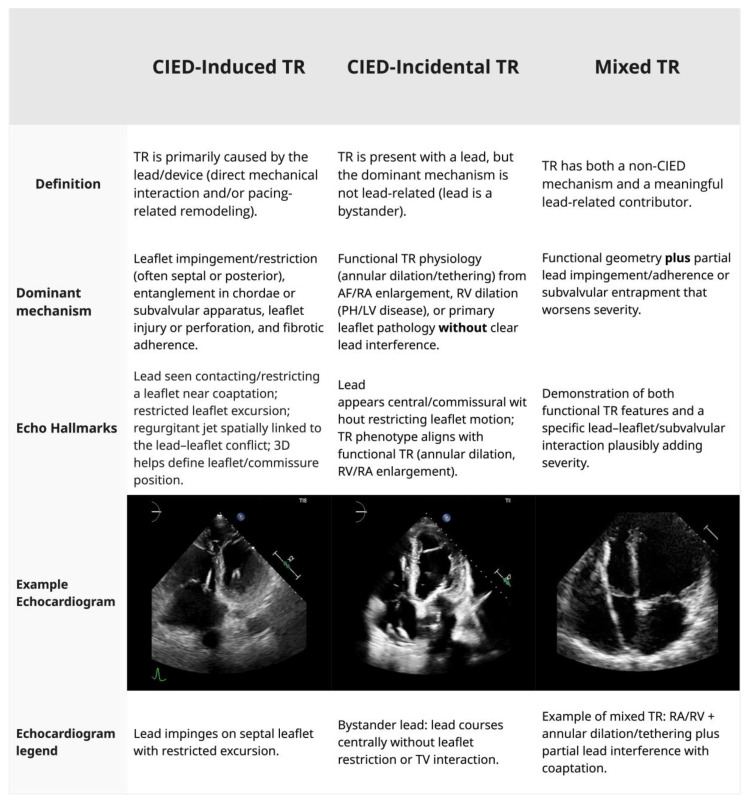
Phenotypes of tricuspid regurgitation (TR) in patients with cardiac implantable electronic device (CIED) leads and distinguishing CIED-induced, CIED-incidental, and mixed TR. Abbreviations: 3D, three-dimensional; AF, atrial fibrillation; CIED, cardiac implantable electronic device; LV, left ventricle; PH, pulmonary hypertension; RA, right atrium; RV, right ventricle; TR, tricuspid regurgitation; TV, tricuspid valve.

Tricuspid regurgitation (TR) in patients with cardiac devices is a complex clinical scenario, particularly as transcatheter tricuspid valve interventions (TTVI) have become more common, with 12% to 36% of patients evaluated for TTVI having a transvalvular pacemaker or defibrillator lead present [1]. The presence of a CIED lead across the tricuspid valve can be causally related to TR (CIED-induced TR) or a bystander (CIED-incidental TR), with the lead lying across the valve without impeding leaflet coaptation (Figure 1). The mechanisms of CIED-induced TR are: (1) mechanical interference from the lead, (2) leaflet perforation, (3) chordal damage, (4) pacing-induced RV remodeling, (5) device-related infections resulting in valve destruction from endocarditis, and (6) fibrosis involving the lead and TV [2,3,4]. True CIED-induced TR occurs in 5–7% of patients with clinically significant TR [5]. The presence of CIED leads can result in the progression of TR in 7.2% to 44.7% of cases [6,7,8,9]. Distinguishing passive coexistence from CIED-induced TR is critical, as it has implications for management. Moderate or severe TR in the presence of CIED confers a markedly worse prognosis, with a 1.6- to 2.5-times increased risk of death [10,11].

**Figure 2:**
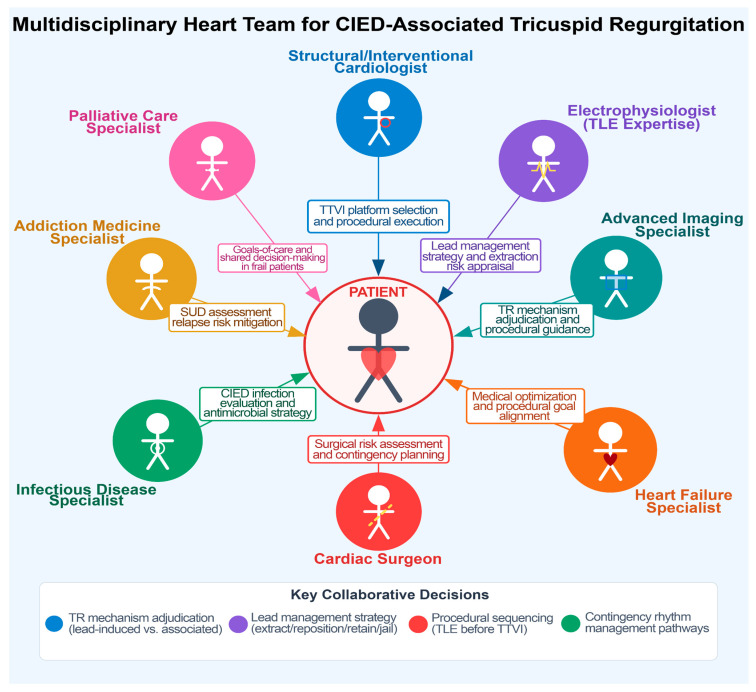
Proposed composition of a Heart Team managing patients with transvalvular right ventricular leads undergoing transcatheter tricuspid valve interventions. Abbreviations: TLE: transvenous lead extraction; CIED: cardiac implantable electronic device; TR: tricuspid regurgitation; TTVI: transcatheter tricuspid valve intervention; TTV: transcatheter tricuspid valve; SUD: substance use disorder.

The management of TR in the presence of transvalvular CIED poses unique challenges. The presence of a lead across the valve interferes with transcatheter therapies, raising additional procedural considerations: (1) jailing of a lead between a prosthetic valve or annular device and the native anatomy may damage the lead or impede the valve intervention; (2) entanglement or dislodgement of the lead may occur during catheter manipulation; (3) extracting a chronic pacing lead to avoid jailing prior to TTVI carries procedural risks such as lead fracture, embolization, and need for emergency surgery; and (4) careful decision-making is required to decide if, and what type of, CIED that spares TV needs to be implanted. The complexity of these decisions is ideally addressed by multidisciplinary Heart Teams (Figure 2, Table 1) that carefully weigh the trade-offs of each individual decision [12,13].

This review synthesizes the available evidence from registries, observational studies, and recent expert consensus statements to provide a practical framework for Heart Teams managing this increasingly common complex clinical scenario. We highlight the critical knowledge gaps and propose directions for future investigations. Currently, no randomized clinical trials (RCTs) have compared management strategies in this population, and clinicians must navigate complex trade-offs, such as lead extraction versus jailing, repair versus replacement, and alternative pacing strategies, with limited long-term outcome data available. This interdependence of rhythm-management and valve-therapy strategies represents the “two-device problem,” wherein decisions about CIED management are inseparably linked to the feasibility, safety, and durability of tricuspid valve interventions, creating an urgent need for consolidated guidance.

## 2. Methodology

We conducted a narrative review to provide a comprehensive synthesis of the existing literature on CIED-related TR and its management in the context of transcatheter tricuspid valve interventions. A targeted literature search was performed in PubMed/MEDLINE and Embase supplemented by manual review of references from key articles and relevant society documents to identify clinical studies, registries, device reports, and consensus statements addressing TR in CIED populations and/or lead management in the context of TTVI. Search terms included combinations of tricuspid regurgitation, pacemaker, implantable cardioverter-defibrillator, cardiac implantable electronic device, transcatheter tricuspid, tricuspid edge-to-edge repair, TriClip, PASCAL, transcatheter tricuspid valve replacement, EVOQUE, annuloplasty, Cardioband, caval valve implantation, valve-in-valve, lead extraction, and lead jailing.

We prioritized (i) randomized trials when available; (ii) prospective and retrospective observational studies and registries; (iii) device-specific reports relevant to lead–TTVI interactions; and (iv) major professional society guidelines/consensus statements addressing TTVI, CIED lead management/extraction, and valve-sparing pacing/defibrillation options. We focused on adult human data and included studies in English. We excluded studies not pertinent to transvalvular lead–tricuspid valve interaction and reports where outcomes could not be reasonably attributed to the CIED–tricuspid interface.

## 3. Mechanisms of Tricuspid Regurgitation in Patients with CIEDs and Diagnostic Evaluation

The mechanisms by which CIED leads contribute to TR are categorized as (1) direct mechanical interference, including leaflet impingement, perforation, chordal entanglement, and fibrotic adhesion, and (2) functional TR, from RV pacing-induced dyssynchrony and/or remodeling, leading to annular dilation and papillary muscle displacement. Understanding these mechanisms is essential for distinguishing CIED-induced TR from CIED-incidental TR (Figure 1), a distinction that directly informs management strategies.

### 3.1. Mechanical Interference

The mechanical interaction between CIED leads and the TV is the most common mechanism of CIED-induced TR. The septal leaflet is most frequently affected [3]. CIED-induced injury may occur at the time of implantation or evolve chronically over the years. Acute TR is rare and occurs due to leaflet perforation, damage to the chordae or papillary muscles, resulting in flail leaflet physiology and rapid clinical deterioration [14,15]. Lead impingement can prevent adequate leaflet coaptation, resulting in eccentric regurgitant jets that are often underestimated on standard two-dimensional imaging. Over time, repetitive motion and chronic inflammation may lead to fibrous encapsulation of the lead, tethering of the leaflet or subvalvular apparatus, and further exacerbation of TR [16].

### 3.2. Functional TR

CIED leads can be causally related to TR through pacing-induced structural remodeling and alterations of RV function. Chronic RV pacing alters the normal sequence of ventricular activation, leading to mechanical dyssynchrony and ultimately promoting RV dilatation. This, in turn, results in TV annular enlargement, apical displacement of RV papillary muscles, and tethering of the TV chords, causing functional TR even in the absence of direct lead–leaflet interaction [17]. Although there is no consensus on the RV pacing threshold that increases the risk of developing functional TR, a higher RV pacing burden is associated with a higher risk of developing RV dysfunction and functional TR. Pacing-induced left ventricular systolic dysfunction may further worsen TR severity by increasing pulmonary pressure and RV afterload [17,18].

### 3.3. Diagnostic Evaluation: Multimodality Imaging

Accurate characterization of the TR mechanism in patients with CIEDs relies on a multimodality imaging approach.

#### 3.3.1. Echocardiography

Two-dimensional and three-dimensional transthoracic echocardiography (TTE) are the primary diagnostic modalities for evaluating TR severity, mechanism, and right-sided chamber remodeling [19,20]. Three-dimensional echocardiography is particularly valuable for defining the spatial relationship between the lead and the TV apparatus, allowing visualization of leaflet impingement, restricted motion, or commissural versus central lead position [21]. Leads positioned near the commissures or the middle of the annulus are generally associated with less leaflet interference than those coursing directly over the leaflet bodies [22].

Transesophageal echocardiography (TEE) provides higher spatial and temporal resolution and is often required for definitive mechanism classification, particularly when planning transcatheter intervention or transvenous lead extraction (TLE). TEE enables a detailed assessment of leaflet perforation, adhesions, or flail segments and plays a central role in intraprocedural guidance during TTVI [22,23,24]. Standardized TR quantification, integrating vena contracta width, effective regurgitant orifice area, hepatic vein flow, and three-dimensional jet assessment, is essential to avoid underestimation of the severity of CIED-induced TR [19,20].

#### 3.3.2. Computed Tomography

Cardiac CT offers superior spatial resolution and is increasingly used for procedural planning in patients with CIEDs undergoing TTVI or transvenous lead extraction (TLE). CT allows precise assessment of lead–leaflet interaction, tricuspid annular dimensions, and proximity to adjacent structures such as the right coronary artery, which is critical for transcatheter valve replacement planning. CT characterizes central venous anatomy, which is relevant in patients being considered for extraction or CIED implants that do not include transvalvular leads [25].

## 4. Transcatheter Tricuspid Valve Interventions and CIED Lead Considerations

**Figure 3:**
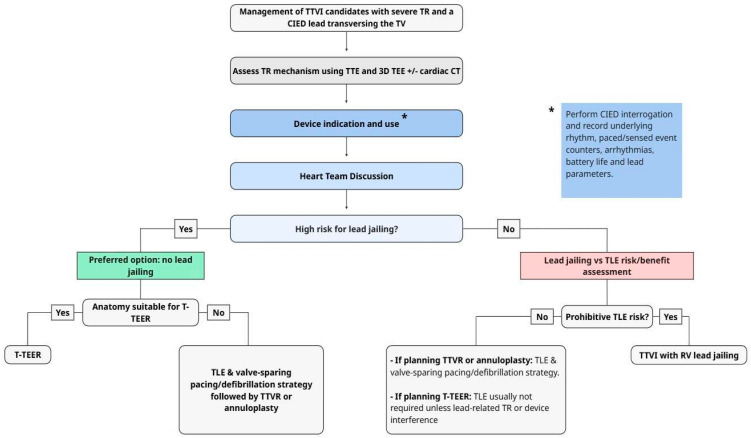
Proposed algorithm for the management of TTVI candidates with symptomatic severe TR and a CIED lead crossing the TV, adapted from the 2025 ECAVI/EHRA scientific statement on Management of patients with transvalvular right ventricular leads undergoing transcatheter tricuspid valve interventions. Abbreviations: 3D: three-dimensional; CIED: cardiac implantable electronic device; CT: computed tomography; RV: right ventricular; T-TEER: transcatheter tricuspid edge-to-edge repair; TEE: transesophageal echocardiography; TLE: transvenous lead extraction; TR: tricuspid regurgitation; TTE: transthoracic echocardiography; TTVI: transcatheter tricuspid valve intervention; TTVR: transcatheter tricuspid valve replacement; TV: tricuspid valve.

Current TTVI modalities include (1) transcatheter edge-to-edge repair (T-TEER) using devices such as TriClip and PASCAL; (2) transcatheter tricuspid valve replacement (TTVR) with systems such as EVOQUE; (3) direct percutaneous annuloplasty (Cardioband); (4) heterotopic caval valve implantation (CAVI); and (5) tricuspid valve-in-valve (TTVIV) procedures for failed surgical bioprostheses. Understanding how each TTVI modality interacts with existing CIED leads is essential for procedural planning and lead management. This section reviews the current TTVI strategies and the lead-related risks associated with each approach (Table 2, Figure 3).

### 4.1. Transcatheter Tricuspid Edge-to-Edge Repair (T-TEER)

T-TEER is the most widely adopted TTVI modality due to its favorable safety profile and the ability to maintain future therapeutic options. T-TEER approximates the tricuspid leaflets to reduce the regurgitant orifice area, mimicking the surgical Alfieri stitch. Currently available T-TEER devices are the TriClip [26] (Abbott Structural Heart) and PASCAL [27] (Edwards Lifesciences, CE-marked in the EU, but not FDA-approved yet). In the TRILUMINATE trial, T-TEER was superior to medical therapy alone in reducing heart failure hospitalizations and improving quality of life and patient-reported health status [28].

T-TEER is the preferred first-line TTVIs strategy for patients with CIED. This allows for the preservation of lead mobility and does not mandate the fixation of the lead against annular tissue. T-TEER preserves future lead-management options. T-TEER does not jail the lead in a manner that precludes future TLE, and standard TLE tools can be advanced alongside the clips. Furthermore, the presence of a transvalvular lead was not associated with any differences in the safety and effectiveness of T-TEER, the degree of residual TR, and or the one-year mortality in the TRIUMINATE trial and the post-approval TriValve Registry [28,29].

A critical decision that needs to be made during the planning of T-TEER in a patient with TR and CIED leads is whether the patient has CIED-induced TR or if the leads are innocent bystanders. In most cases suitable for T-TEER, the lead is an “innocent bystander.” The lead typically lies in a commissural position or sits freely within the regurgitant orifice without tethering the leaflets [30]. In these cases, T-TEER is feasible, and the clips are deployed at a distance from the lead, ensuring that the grasping elements do not capture the lead body. Conversely, in CIED-induced TR, the presence of leaflet impingement, chordal entanglement, or fibrous adhesions makes T-TEER technically more challenging. In these patients, device deployment close to or on the lead might be necessary, in which case there is a risk of damaging the lead or causing lead jailing, requiring a multi-disciplinary approach [12,30].

With the use of intraprocedural 3D TEE, the risk of T-TEER and the risk of grasping a transvalvular lead are exceedingly low (<1%). If, however, a lead is inadvertently captured, the clipping elements can penetrate the lead insulation or crush the internal conductors, causing lead failure [31].

### 4.2. Transcatheter Tricuspid Valve Replacement (TTVR)

TTVR corrects TR through orthotopic implantation of a bioprosthetic valve. The only currently approved device is EVOQUE (Edwards Lifesciences). Both rely on radial force and varying anchoring mechanisms to be secured within the native annulus. TTVR offers superior efficacy in correcting TR compared to T-TEER, with near-complete elimination of TR [32]. In the TRISCEND II RCT, the EVOQUE valve was associated with a significant improvement in 1-year all-cause mortality, functional status, and quality of life. However, given the proximity of the conduction system to the tricuspid annulus and the radial force exerted by the self-expanding frame of the EVOQUE valve, complete heart block requiring pacemaker implantation can occur in up to one-third of the implants [33,34]. This significant risk is a critical consideration for patient selection, particularly in those with RV dysfunction, where dyssynchronous pacing can be deleterious [17].

In TTVR, lead jailing is an unavoidable procedural consequence in any patient with a pre-existing transvenous lead [1]. Historically, “metal on tissue jailing” was hypothesized to be relatively benign for jailed leads [35]. However, emerging data challenge the perceived safety of metal-on-tissue jailing. In a retrospective analysis of 32 patients undergoing EVOQUE implantation with a jailed lead, 31% developed lead malfunction during a median follow-up of 210 days, including a decline in R-wave sensing and insulation breach (13% of participants). While only one patient required immediate lead revision in this series, the long-term implications of jailing remain unknown [36]. As a result, it is common practice for the multidisciplinary TTVR team discussion to involve electrophysiology for consideration of non-transvalvular pacing options discussed in Section 4.5. Last, the most profound implication of TTVR for CIED leads is the permanent preclusion of transvenous lead extraction. Once a lead is jailed behind a TTVR device, it cannot be safely extracted using standard mechanical or laser tools without the risk of avulsing the entire valve complex or tearing the annulus. Careful multidisciplinary evaluation and shared decision-making to proceed with jailing or with TLE prior to TTVR is critical and will be described in Section 4.

### 4.3. Direct Percutaneous Annuloplasty

Direct percutaneous annuloplasty is a reconstructive approach that replicates surgical suture annuloplasty. The Cardioband system (Edwards Lifesciences) is the only clinically available device. It has CE-mark approval in Europe but has not received FDA approval in the United States, where use remains investigational in clinical studies [37]. The system consists of a Dacron band anchored along the tricuspid annulus using a series of stainless-steel screws and cinched to reduce annular dimensions. In limited RCTs, the Cardioband system results in significant improvement in TR and patient functional status [38].

The interaction profile of the Cardioband system and CIED leads differs from that of T-TEER and TTVR. The interaction mechanism is a direct mechanical collision between the device anchors and transvalvular lead. Transvenous leads typically cross the tricuspid valve in the septal region, often settling in the posteroseptal or anteroseptal commissures. This overlaps with the anchoring zone of the Cardioband along the anterior and posterior annulus. There is a theoretical risk that a Cardioband system anchor penetrates the lead body, causing insulation failure and/or conductor fracture [39]. Even in the absence of direct lead injury from the anchoring system, as the band is cinched to reduce the annular circumference, the lead can be pulled medially or compressed against the septal wall, forming a fulcrum point on the lead, potentially accelerating mechanical failure. While Cardioband does not create a circumferential cage like TTVR, it can effectively jail a lead if the band is cinched tightly against it. However, unlike TTVR, lead extraction remains theoretically feasible, provided that the lead has not been skewered by an anchor.

### 4.4. Caval Valve Implantation (CAVI)

CAVI is a palliative strategy that involves the implantation of bioprosthetic valves into the superior vena cava (SVC) and inferior vena cava (IVC). Among dedicated CAVI platforms, the TricValve system (P&F Products and Features) is CE-marked and available in Europe, whereas U.S. use remains investigational under studies. In addition, balloon-expandable transcatheter heart valves (e.g., Edwards SAPIEN XT/3) have been used for intracaval implantation, typically after creation of a landing zone with a large stent, but this is off-label/investigational repurposing rather than an approved CAVI indication for SAPIEN. CAVI prevents systolic backflow and alleviates systemic venous congestion. CAVI is reserved for patients with severe TR, massive annular dilation (>65–70 mm), who are not candidates for transcatheter repair or replacement, and are at prohibitive surgical risk. CAVI improves ascites, peripheral edema, functional class, and, in limited studies, reverses the remodeling of the right ventricle [40,41].

CAVI presents a unique challenge from a lead interaction perspective: the entrapment of the lead in the superior vena cava. The implantation of a stent valve in the SVC jails all transvenous leads behind the stent mesh along the entire length of the SVC. Lead dislocation during CAVI has been previously described [42]. The superior extent of the SVC valve often reaches or covers the brachiocephalic vein confluence. This creates a mechanical barrier to future implantations. Extracting a lead that is jailed behind an SVC stent is high-risk because the extraction sheath must traverse the stent, with a high risk of stent dislodgment, SVC laceration, or lead fracture. The decision to proceed with CAVI in a patient with transvenous leads should be made after careful multidisciplinary Heart Team assessment and shared decision-making, similar to TTVR.

### 4.5. Tricuspid Valve-in-Valve (TTVIV)

TTVIV procedures use transcatheter valves to address degenerated surgical bioprosthetic valves. Most commonly used platforms are the Medtronic Melody and Edwards Sapien. TTVIV is an alternative to high-risk redo surgery for bioprosthetic TV failure, with high procedural success and low mortality [43]. Importantly, although these transcatheter valves are commercially available in the United States and Europe for their labeled indications (SAPIEN for aortic position, Melody for pulmonic position), use in the tricuspid position is typically off-label and performed under Heart Tteam and institutional governance rather than a tricuspid-specific regulatory indication. TTVIV has the same implications for transvalvular CIED leads with TTVR, with the addition that in TTVIV, there is a more hostile mechanical environment for the lead due to the presence of a rigid surgical frame as opposed to native annular tissue. This results in a “Metal-on-Metal” jailing mechanism. Leads jailed between these two valves or between a valve and a ring are subjected to extreme focal compression and shearing forces that can rapidly sever lead insulation and/or fracture the conductors. In the VIVID registry, the lead failure rate was 10.7% over a median follow-up of only 15.2 months in patients with jailed leads in the setting of TTVIV [44,45]. This failure rate is significantly higher than that observed in native valve TTVR or T-TEER [43,44]. We suggest that the presence of a functional lead is a strong relative contraindication to TTVIV. TLE and replacement in favor of a new leadless or epicardial system should be strongly considered in such cases.

## 5. CIED Lead Management Strategies

### 5.1. Patient Assessment

#### 5.1.1. Assessment of Transvenous Lead Extraction Risk

The risk associated with TLE is integral to extraction–preservation decision-making. Risk assessment for TLE should distinguish (i) predictors of major procedure-related complications/procedural death from (ii) predictors of short-term mortality and long-term prognosis after an otherwise successful extraction. Patient factors such as low body mass index, anemia, coagulopathy, and frailty more consistently track with procedural vulnerability/bleeding reserve, whereas comorbidity burden (e.g., renal dysfunction, reduced LVEF, multimorbidity) more strongly influences early and late mortality rather than the mechanical complication risk of extraction itself [46,47,48]. Lead-related factors include prolonged dwell time (typically >10–15 years for pacemaker leads and >5–10 years for ICD leads), multiple transvenous leads, dual-coil ICD leads, fixation mechanism, and insulation characteristics [47,48,49]. Contemporary data from high-volume centers report major complication rates of approximately 1.7% and mortality around 0.5%, with outcomes strongly correlated with operator experience and institutional volume [47,48]. To put this risk into perspective, the complication rates and mortality risks at 30 days from TAVR are 9–12% and 2.2–2.4%, respectively [50]. Thus, it is a common misconception that the risk of TLE is extremely high. We support inclusion of the electrophysiology team as part of the Heart Team evaluating patients with CIED leads undergoing TTVI evaluation at centers with high-volume experience in TLE. Importantly, contemporary analyses show that advanced age alone is not an independent predictor of major TLE complications when procedures are performed in experienced centers. Conversely, some datasets have reported higher major complication risk in younger patients/younger age at first implant, likely reflecting longer cumulative dwell time and more complex lead biology, reinforcing that chronological age should not be used as a reflex disqualifier [51].

#### 5.1.2. Assessment of Lead Jailing Risk

The risks of lead jailing are categorized as (1) risks related to the lead and (2) risks related to the prosthetic transcatheter valve. The risks related to the lead are as follows: (1) pacemaker dependency, as lead failure in a pacemaker-dependent patient could have catastrophic consequences; (2) high infectious risk (see Section 5.1.3), as TLE of a jailed infected lead would be of extremely high risk and even not possible; and (3) history of appropriate ICD therapies, as this selects a higher risk population for the development of life-threatening arrhythmias, in which case a failing lead will be ineffective in delivering successful cardioversion/defibrillation. Risks related to the valve include (1) multiple transvenous leads crossing the tricuspid valve, (2) high lead tension with minimal slack, and (3) established leaflet impingement, particularly in the context of direct annuloplasty systems [35,44]. In the presence of these features, lead jailing may irreversibly compromise life-sustaining device therapy and preclude future extraction, and is therefore discouraged in favor of TLE [12,44].

#### 5.1.3. Assessment of CIED Infection Risk

Risk stratification for CIED infection is a critical adjunct to lead management decisions, particularly when lead preservation or jailing is considered. Factors associated with increased CIED risk factors are summarized in Table 3 [46,47]. In patients with high infection risk, the consequences of lead jailing are significant, as standard management of CIED infection requires complete system removal, which may be prohibitively risky and even technically not possible in jailed leads [35,46].

### 5.2. Transvenous Lead Extraction as a Standalone Strategy

Transvenous lead extraction (TLE) can improve tricuspid valve function in a subset of patients with CIED-induced TR. Improvement in tricuspid valve function after extraction was observed in approximately one-third of cases [52]. TLE alone does not cure TR, specifically if TV annular dilatation has occurred or if the TV leaflet has adhered to the annulus. TLE as a standalone intervention for TR can be considered in cases where there is (1) unequivocal imaging data that show that the lead–leaflet interaction is the primary driver of TR, (2) absence of significant annular dilatation, and (3) absence of valve adhesions to the annulus or RV that limit mobility. Even in these cases, shared decision-making should include the roughly 1/3 chance of meaningful TR improvement with TLE alone and the possibility of worsening TR requiring urgent valvular intervention (percutaneous or surgical). However, TLE should be avoided when procedural risk clearly outweighs potential benefit, such as in patients with extreme frailty or prohibitive comorbidities, in the presence of hostile lead anatomy (severe calcification, long dwell times, multiple ICD leads, or abandoned leads), when surgical backup is limited, or when patient preferences favor a conservative strategy.

Rigorous risk stratification is essential for TLE shared decision-making, requiring distinction between factors predicting procedural complications versus long-term mortality. High-risk extraction features that most consistently predict major procedure-related complications include hostile lead/anatomic factors (e.g., long dwell time, multiple/abandoned leads, ICD leads, severe fibrosis/calcification, venous occlusion) and limited institutional readiness for rescue. In contrast, advanced age, renal dysfunction, reduced LVEF, and multimorbidity primarily predict early/late mortality after TLE and should be integrated into overall benefit–risk and goals-of-care discussions rather than treated as direct surrogates of extraction mechanical hazard [46].

### 5.3. TLE and Lead Preservation/Jailing in Conjunction with Percutaneous Tricuspid Valve Intervention

TLE followed by TTVI can be considered in three settings: (1) TTVI procedures that will result in CIED lead jailing, (2) TTVI procedures that do not result in jailing, and (3) TTVI procedures as “rescue” procedures for worsening TR after TLE. For TTVI that will result in CIED jailing, the authors strongly recommend TLE over jailing the lead and jailing only if there are absolute contraindications or extreme technical difficulties to TLE. Elective TLE mitigates the permanent problem of an entrapped lead segment that is difficult or impossible to extract later, including in the event of device infection [46,50]. Although there is concern that TLE could cause traumatic tricuspid apparatus injury (e.g., leaflet avulsion/flail) that might compromise feasibility of subsequent T-TEER or TTVI, contemporary consensus data suggest this is uncommon in experienced programs: the 2018 EHRA consensus reports flail tricuspid leaflet requiring intervention in ~0.03% of cases, with worsening tricuspid valve function in ~0.02–0.59% [53]. For repair strategies, the advantage of extraction is less uniform and should be decided on a case-by-case basis. In certain scenarios, TLE has an additive benefit, such as when it is performed to eliminate leaflet restriction that would impair coaptation after TEER [46]. Of note, TTVI are typically planned elective procedures for chronic symptomatic TR, timing of TLE is flexible: the valve intervention can be postponed to allow (i) referral to an experienced extraction center when TLE risk/expertise warrants and/or (ii) a staged strategy in which TLE is performed days–weeks before (rather than during) transcatheter intervention when clinically appropriate. We recommend that jailing is strongly avoided and is performed only when there is a strong justification based on a multidisciplinary risk–benefit analysis (Figure 3). Jailing allows near-term procedural simplicity but introduces lifetime formidable challenges in lead management, particularly in pacing-dependent patients, those with prior appropriate ICD therapy, or those at high risk for infection [46].

When lead jailing is contemplated, the following need to be strongly considered and presented to the patient in a shared-decision-making framework: (1) Jailed leads are at risk for lead failure due to insulation breach and conductor fracture, resulting in loss of defibrillation/pacing reliability [35,44,45]. These risks are incompletely quantified because follow-up is limited and cohorts remain small; however, worsening of electrical parameters after TTVR has been reported, including the need for lead revision [35,45]. (2) Future TLE may be higher risk and even impossible once a lead is entrapped. If any pocket, device-related, or endovascular infection occurs, the standard of care is complete system removal, and the patient may not have access to a life-saving procedure.

### 5.4. Pacing and Defibrillation Strategies in Patients with TTVIs After Extraction or Jailing

After TTVI is completed, regardless of whether the existing leads are extracted or jailed, a decision should be made regarding the new pacing or defibrillator system that needs to be implanted. First, it is critical to reassess the need for a pacemaker or ICD. In a large observational cohort, ~84% of patients with implanted pacemakers were not pacemaker-dependent on follow-up (i.e., 16% pacing-dependent) [54]. Approximately 25% of patients with an ICD for primary prevention no longer meet the indications for primary prevention ICDs [55]. In these patients, device reimplantation may be deferred. Conversely, following orthotopic TTVR, patients may develop worsening or de novo conduction disease necessitating pacemaker implantation. In TRISCEND II (Evoque), 17.4% of patients required a new permanent pacemaker.

Second, for patients who need a pacemaker or ICD, the risk of having a pacemaker or ICD lead across the newly implanted device needs to be conceptualized. There is limited and conflicting evidence on whether having a lead across a prosthetic tricuspid valve is deleterious to valve function [52,56,57]. Third, for patients who need CIED reimplantation and a transvalvular lead is not desired, the optimal non-transvalvular system needs to be selected, weighing the risks and benefits of each option, together with the patient’s preferences.

**Figure 4:**
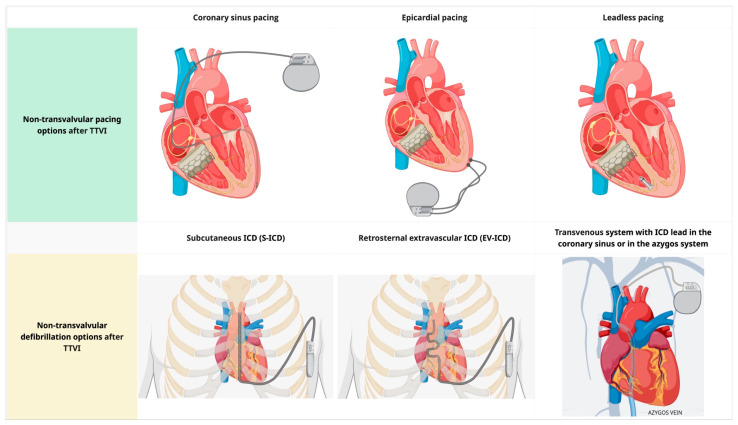
Non-transvalvular pacing and defibrillation options after transcatheter transvalvular interventions. Abbreviations: TTVI: transcatheter tricuspid valve intervention; ICD: implantable cardioverter-defibrillator; S-ICD: subcutaneous implantable cardioverter-defibrillator; EV-ICD: extravascular implantable cardioverter-defibrillator.

For patients who require right ventricular pacing, non-transvalvular pacing options include (1) coronary sinus (CS) pacing, (2) a leadless pacemaker (MICRA or AVEIR), and (3) epicardial pacing (Figure 4). CS pacing is an option in patients in whom crossing the tricuspid valve is undesirable or impossible, with acceptable long-term lead performance [58,59]. CS-only ventricular pacing can be limited by higher capture thresholds, phrenic nerve stimulation, lead instability, a higher risk of acute dislodgment compared to RV leads, and challenges in achieving reliable sensing/capture in anatomically constrained venous targets. Leadless pacemakers can overcome some limitations of CS-only ventricular pacing. Implantation of an RV leadless pacemaker requires crossing the prosthetic valve with a large-caliber delivery system at the time of implantation, but leaves no lead across the tricuspid valve. The implantation of leadless pacemakers can be safely performed in patients with prosthetic tricuspid valves. The Micra system is smaller than AVEIR and may be preferred to reduce device–catheter interaction during valve deployment; however, Micra AV provides accelerometer-based atrial mechanical sensing without atrial pacing, and therefore does not offer ‘true’ AV synchrony. Leadless pacemakers should be strongly preferred in the setting of high infection risk or limited vascular access [60]. Long-term real-world follow-up of leadless pacemakers demonstrates low rates of major complications and system revisions, with an extremely low incidence of infection [61]. Epicardial pacing systems for single-site ventricular pacing should be considered in the extremely rare setting where the other options presented here are not feasible.

For patients who need ICD, non-transvalvular pacing options are: (1) subcutaneous ICD (S-ICD), (2) retrosternal extravascular ICD (EV-ICD), and (3) transvenous system with ICD lead in the coronary sinus or in the azygos vein (Figure 4). The latter is associated with a high risk of extraction and should be used judiciously [62]. Both S-ICD and EV-ICD are non-inferior to transvenous systems in providing adequate cardioversion/defibrillation [63,64]. The main advantages of the EV-ICD over the S-ICD are the smaller generator size, longer battery longevity, and ability to deliver ATP [65]. This comes with the trade-off of a more complex implantation procedure and a higher risk of over- or undersensing [65]. Prior sternotomy is a relative contraindication for EV-ICD implantation. Neither S-ICD nor EV-ICD can deliver pacing for bradycardia indications; if pacing is also required, a hybrid approach with implantation of a leadless device (as described in the previous paragraph) can be combined with the S-ICD and EV-ICD. Caution should be exercised to prevent device–device interactions that result in inappropriate sensing. The presence of atrial pacing is a contraindication for EV-ICD due to the risk of inappropriate sensing, resulting in inappropriate shock [63]. A modular leadless pacemaker that can be combined with the S-ICD is currently available in limited market release (EMPOWER leadless pacemaker, Boston Scientific) [66].

For patients who require cardiac resynchronization therapy, the only option that spares the tricuspid valve is total epicardial or hybrid epicardial (RV) combined with transvenous (RA and CS) pacing systems. Epicardial systems are also a salvage approach for patients without vascular access. Placement of an epicardial system has its own technical challenges and surgery-related morbidity [67,68]. Epicardial leads have a significantly higher risk of lead failure and worsening lead parameters over time than endocardial leads [69,70]. These need to be weighed carefully against the controversial risk of having a single lead across a bioprosthetic tricuspid valve. In our institutions, we prefer an endovascular CRT system over an epicardial system for patients with TTVI who require CRT, provided that the risk of infection is low.

## 6. Management of Jailed CIED Leads

### 6.1. Follow-Up of Patients with CIED and Jailed Leads

Given the potential for accelerated lead failure in the setting of a jailed CIED lead, structured post-procedural surveillance is mandatory [35,44]. Remote monitoring should be activated whenever available, with alert pathways configured for impedance deviations, threshold rises, sensing drops, noise/oversensing, mode switches, and ICD therapy events [71,72].

In-person follow-up frequency after jailing is not supported by high-quality comparative trials and therefore should be framed explicitly as expert-consensus practice [35,44,73]. For patients without remote monitoring capability, in-office evaluations every 3 months during the first year and every 3–6 months thereafter are reasonable, with closer evaluations in pacemaker-dependent patients or those with prior appropriate ICD therapy [44,73]. Any new device alert, abrupt parameter change, recurrent oversensing/noise, inappropriate therapies, or symptomatic deterioration should trigger expedited assessment [46]. If lead failure is confirmed, management decisions should occur within a Heart Team framework, given the added complexity conferred by a jailed lead [44,46].

### 6.2. Management of Lead Malfunction in Jailed Leads

Lead management in failing jailed leads follows the same principles as non-jailed leads, with special considerations that (1) TLE is of considerably higher risk and complexity and might not even be feasible; (2) crossing the prosthetic valve has a theoretical risk of causing valve dysfunction, although data for the latter are limited and conflicting; and (3) the presence of a transvalvular lead might compromise future valve re-interventions [48,52,56]. If a lead malfunction is detected in a CIED with a jailed lead, it must first be determined whether the patient still needs the function of the failing lead [54,55]. If the lead function is still needed, first-line management should be conservative with device reprogramming (output/sensitivity adjustment, optimization of noise discrimination algorithms). In the setting of an ICD, defibrillation threshold or margin testing can be considered to assess the ability of the ICD to cardiovert patients in real life. It is critical to assess the impact of programming changes on battery longevity, considering patients’ survival expectancy, as multiple generator changes exponentially increase the risk of infection. If reprogramming cannot restore meaningful lead function with reasonable battery longevity, then the addition of a new pacing or ICD system with abandonment is generally preferred, given the increased risk associated with TLE of jailed leads, even in experienced centers, and the optional nature of the latter [74]. When a new pacing or ICD system is pursued, valve-sparing options described in Section 4.5 are preferred, due to concerns about the need for future valve reinterventions [45,74,75].

### 6.3. Management of CIED and Endovascular Infections in the Setting of Jailed Leads

Infection management in patients with jailed leads is complex. First, the desired scope of treatment (curative vs. palliative) should be decided based on patient goals of care, frailty, comorbidity, and operative candidacy [76,77,78]. Second, the type of infection should be determined: (1) isolated pocket infection, (2) bloodstream infection without definite tricuspid valve device involvement, or (3) lead or valve endocarditis with tricuspid involvement [76,78]. If the scope of treatment is curative, in the absence of TV involvement, complete device removal should be pursued; however, with jailed leads, a TLE may be considered only in carefully selected patients at experienced centers after Heart Team review [49,78]. TLE in this setting is technically challenging and, in some cases, impossible because traction and powered sheaths transmit force to the prosthesis and annulus, increasing the risk of valve damage, paravalvular leak, cardiac perforation, and lead breakage [44]. TLE has been described in jailed leads in case reports [79]. When infection involves the prosthetic tricuspid valve, the curative approach is surgical explant of the tricuspid device plus complete CIED removal [79].

If the scope of treatment is palliative, long-term suppressive antibiotic therapy can be used for endovascular infections. The prognosis of chronic suppressive antibiotics is less favorable if complete source control is not achieved [80]. For pocket infections, local ultrahigh-dose antibiotic administration remains investigational and should be framed as a salvage strategy for selected localized pocket infections in patients unsuitable or unwilling to undergo extraction [81].

## 7. Heart Team Approach and Workflow

The unique “two-device” problem created by transvalvular leads in patients undergoing TTVI necessitates a formal multidisciplinary Heart Team assessment integrating valve disease decision-making with lead management expertise [12,44]. This multidisciplinary approach is essential because procedural strategy selection (percutaneous vs. surgical and type of TTVI) directly influences current and future rhythm-management options. Decisions regarding lead extraction, repositioning, retention, or jailing have durable implications for pacing dependency, defibrillation strategy, infection risk, and venous access complications. The Heart Team should include a structural valve specialist, cardiac electrophysiologist, advanced imaging specialist (with intraprocedural experience), heart failure clinician, and cardiac surgeon [12]. Infectious disease, addiction medicine, and/or palliative care specialists can be included when indicated (Table 1, Figure 2). Key decisions include (1) accurate characterization of the TR mechanism (CIED-induced vs. CIED-associated), (2) optimal TTVI modality, (3) extraction vs. lead preservation risk appraisal, (4) procedural planning, especially if concomitant TLE and TTVI procedures are included, and (5) pre-specification of contingency CIED pathways that avoid chronic transvalvular hardware, when feasible.

## 8. Current Guidelines

### 8.1. EHRA/EAPCI 2025 Scientific Statement on Management of Patients with Transvalvular Right Ventricular Leads Undergoing Transcatheter Tricuspid Valve Interventions

The 2025 EHRA/EAPCI 2025 statement on the management of patients with transvalvular right ventricular leads undergoing transcatheter tricuspid valve interventions is the only currently available scientific statement that focuses explicitly on the management of patients with transvalvular CIED leads undergoing TTVI [44]. This document highlights the cross-disciplinary scope of the problem and the need for multifaceted assessment and procedure planning. The proposed central algorithm for approaching patients with CIED leads requiring TTVI is summarized in Figure 3. Key recommendations include obtaining complete details of the implanted system prior to any intervention, including assessment of pacemaker dependency and, for ICDs, the type and frequency of tachyarrhythmia therapies delivered. This statement emphasizes a thorough evaluation of TR etiology and TTVI suitability, with particular attention to potential mechanical interactions between CIED leads and transcatheter valve hardware. Central to procedural planning is the assessment of lead jailing risk, for which the statement defines specific “red flags”: pacemaker dependency, ICD with prior delivered therapy, multiple leads crossing the tricuspid valve, previous CIED infection, and high lead tension. Although TLE is recognized as a viable option—particularly in fragile, often elderly patients—it carries a low but non-negligible risk of major complications (1.7%) and death (0.5%) and may paradoxically worsen TR severity in 3.5–15% of cases due to adhesions between leads and the tricuspid apparatus [47,82]. For patients in whom lead jailing is pursued, frequent monitoring is essential, especially for those who are pacemaker-dependent or have an ICD for secondary prevention. Alternative pacing strategies have been proposed to mitigate CIED-induced TR and minimize interactions with implanted tricuspid devices, including coronary sinus ventricular pacing, epicardial pacing, and leadless pacemakers, whereas subcutaneous or extravascular ICDs are recommended for patients requiring defibrillator therapy. Given the limited scientific evidence currently available, the statement underscores that case-by-case Heart Team discussions and patient engagement are essential and calls for prospective systematic data collection and long-term follow-up to strengthen future recommendations [44].

### 8.2. Other Valve or CIED Guidelines Discussing Management of CIED Relevant to Tricuspid Valve Interventions

Beyond the 2025 EHRA/EAPCI scientific statement, several complementary guidelines and expert documents have addressed the intersection of CIEDs and tricuspid valve disease. These are summarized in Table 4.

The 2021 ESC Guidelines on Cardiac Pacing and CRT recognize tricuspid regurgitation as a clinically relevant complication of transvenous RV lead implantation and provide the following recommendations: (1) when pacing is required at the time of tricuspid valve surgery, epicardial ventricular leads should be considered (Class IIa, Level C) to prevent prosthesis interference and future lead–valve conflict; (2) for patients with tricuspid valve prostheses (particularly mechanical valves) in whom transvalvular access is contraindicated, coronary sinus–based or epicardial pacing strategies are acknowledged as valve-sparing alternatives; and (3) structured follow-up pathways, including remote monitoring, are supported to detect device- or lead-related issues early [83].

The 2017 HRS Expert Consensus Statement on CIED Lead Management and Extraction, though predating contemporary TTVI technologies, remains foundational by (1) codifying definitions, procedural endpoints, and outcomes reporting standards; (2) establishing a safety-oriented framework emphasizing that extraction outcomes are tightly coupled to program infrastructure and operator expertise; and (3) mandating institutional preparedness for catastrophic complications—principles that align with the multidisciplinary Heart Team model now advocated for CIED–tricuspid cases [46].

The 2025 ACC/AHA/ASE/HFSA/HRS/SCAI/SCCT/SCMR Appropriate Use Criteria (1) explicitly address the scenario of ventricular pacing indication in patients with prior tricuspid valve surgery and (2) rate leadless pacemaker implantation as “May Be Appropriate” in this setting [84]. This is complemented by the 2022 EHRA/HRS/LAHRS/APHRS Position Paper on Leadless and Extravascular CIEDs, which (1) provides a clinician-facing framework for candidacy and procedural planning, (2) positions leadless pacing as a particularly attractive valve-sparing solution when transvalvular leads are undesirable or infeasible, and (3) outlines considerations for subcutaneous and extravascular ICD systems in patients requiring defibrillator therapy [85].

Collectively, these documents reflect an evolving paradigm in which CIED and tricuspid valve management are no longer considered in isolation but rather as interdependent components of a comprehensive, patient-centered lifetime strategy.

## 9. Future Directions and Research Priorities

Several research priorities must be addressed to advance this field (Table 5). First, multicenter randomized trials or rigorously designed prospective registries with prespecified treatment algorithms are needed to compare extraction versus lead preservation strategies. These studies should have clinically meaningful endpoints, including functional status, heart failure hospitalization, mortality, and device-specific outcomes, such as lead performance, infection, and feasibility of future extraction. Second, the standardization of criteria is essential: unified definitions distinguishing CIED-induced TR from CIED-incidental TR and mixed TR will reduce misclassification and improve cross-study comparability. Third, validated predictive tools are required to identify patients likely to benefit from extraction, stratify procedural risk from pre-procedural imaging, and guide individualized decision-making. Fourth, a dedicated data infrastructure, including international registries with harmonized imaging protocols and jailed-lead cohorts followed for >2 years, is necessary to generate the evidence base required for guideline-level recommendations.

However, a true paradigm shift would be to reimagine the relationship between devices and valves at the earliest stages of patient management, perform CIED implants in such a way that TR is prevented, and develop technologies and techniques that allow for percutaneous lead repair without compromising the leads. TR prevention should be a primary objective, beginning with implantation strategies using real-time 3D echocardiographic guidance to avoid leaflet-interfering trajectories and extending to device selection frameworks that proactively consider lifetime tricuspid valve health. The rapid maturation of leadless and extravascular platforms offers a compelling valve-sparing paradigm: modular systems combining leadless pacemakers with subcutaneous or extravascular ICDs can deliver pacing and defibrillation without transvalvular hardware, whereas emerging leadless conduction system pacing and fully leadless CRT configurations may provide physiologic activation without tricuspid traversal [86]. Next-generation TTVI devices may incorporate lead-accommodating features, such as commissural alignment strategies or dedicated lead channels. Sensor-equipped “smart” leads could detect early valve interaction before clinical TR develops, and artificial intelligence may enable personalized decision-making based on integrated clinical, imaging, and device variables. Ultimately, the goal is a future in which device therapy and tricuspid valve health are managed not as competing considerations but as integrated components of a unified, patient-centered, lifetime strategy.

## 10. Limitations

This review has several limitations inherent to its narrative design. First, although we used a structured search approach, study identification and selection are subject to selection and publication bias. Second, the evidence base informing management of transvalvular leads in the TTVI era remains dominated by observational studies, registries, small series, and expert consensus, with a paucity of randomized comparisons—particularly for extract-versus-jail pathways and long-term outcomes after lead jailing. Last, substantial heterogeneity exists (qualitatively) across studies in TR phenotyping, imaging protocols, device platforms, endpoints, and follow-up duration.

## 11. Conclusions

The management of patients with transvalvular CIED leads undergoing TTVI represents one of the most complex aspects of contemporary cardiovascular medicine. It requires a coordinated Heart Team framework that includes expertise in electrophysiology, structural heart disease, imaging, heart failure, and cardiac surgery. Currently, there is a paucity of randomized evidence to guide management decisions, such as TLE versus lead preservation strategies, limited long-term data on jailed leads, and an incomplete understanding of which patients derive benefit from device modification prior to TTVI. However, this challenge also presents an unprecedented opportunity: the convergence of advancing leadless and extravascular technologies, maturing transcatheter tricuspid platforms, and evolving imaging capabilities creates a path toward a future in which CIEDs and TV disease are managed as integrated components of a unified, patient-centered lifetime strategy rather than competing clinical imperatives. Realizing this vision requires dedicated prospective registries, standardized definitions distinguishing CIED-induced, CIED-incidental, and mixed TR, and collaborative industry–academic partnerships focused on valve-sparing device innovations. As the population of patients with CIEDs and TR continues to grow, so does the urgency—and the promise—of transforming this clinical frontier into an exemplar of precision cardiovascular care.

## Figures and Tables

**Table 1:** Multidisciplinary Heart Team composition and roles.

Team Member	Primary Role	Secondary/Adjunct Role
Structural/Interventional Cardiologist	Procedural candidacy assessment integrating anatomic suitability, frailty, and expected clinical benefit; TTVI platform selection (repair vs. replacement; annuloplasty vs. leaflet-based); procedural execution	Co-ownership of lead-management strategy with EP to minimize lead-related procedural hazards; intraprocedural troubleshooting; preservation of downstream rhythm-management options
Electrophysiologist (TLE expertise)	Lead management strategy (extract/reposition/retain/jail); device interrogation, including rhythm dependence, arrhythmia burden, and ICD therapy history; extraction risk appraisal	Pre-specification of contingency pacing/defibrillation pathways (leadless pacing, CS-based CRT, extravascular ICD); intraoperative CIED programming; post-procedural device surveillance and troubleshooting
Advanced Imaging Specialist	Rigorous TR mechanism adjudication (CIED-induced vs. CIED-incidental); integration of 3D echo and CT for lead–leaflet interaction assessment and anatomical planning	Intraprocedural guidance (device steering, leaflet grasping, complication recognition); coordination of post-intervention imaging surveillance for prosthetic function and hardware interaction
Heart Failure Specialist	Longitudinal clinical context including symptom trajectory, functional status, and HF hospitalization history; medical therapy optimization (diuretics, neurohormonal agents)	Alignment of procedural goals with realistic expectations in comorbid TR populations; post-procedural HF regimen adjustment; long-term surveillance and prognostication
Cardiac Surgeon	Comprehensive surgical risk assessment (redo sternotomy, multivalve disease, endocarditis); surgical backup for procedural complications	Contingency planning when transcatheter options are limited; primary operator role when open intervention is most appropriate; hybrid procedure collaboration
Infectious Disease Specialist	Evaluation of suspected/confirmed CIED infection; antimicrobial strategy development (agent selection, duration)	Coordination with EP regarding extraction timing and reimplantation; long-term suppressive therapy planning; post-procedural infection surveillance
Addiction Medicine Specialist	Substance use disorder assessment and staging; medication-assisted treatment initiation/optimization; relapse risk stratification	Coordination of long-term recovery support and harm reduction; linkage to outpatient addiction services; input on procedural timing relative to recovery stability
Palliative Care Specialist	Goals-of-care discussions; facilitation of shared decision-making when risk–benefit is uncertain; advance care planning, including ICD deactivation discussions	Symptom-focused management in frail/highly comorbid patients; psychosocial support for patients and families; coordination with hospice services when appropriate

Abbreviations: 3D: three-dimensional; CIED: cardiac implantable electronic device; CRT: cardiac resynchronization therapy; CS: coronary sinus; CT: computed tomography; EP: electrophysiology/electrophysiologist; HF: heart failure; ICD: implantable cardioverter-defibrillator; TLE: transvenous lead extraction; TR: tricuspid regurgitation; TTVI: transcatheter tricuspid valve intervention.

**Table 2:** TTVI Strategies and interactions with CIED leads. T-TEER emerges as the most “lead-friendly” TTVI strategy, presenting minimal risk to lead integrity and preserving future options. TTVR, while offering potent TR elimination, is associated with a high risk for the development of complete heart block and creates an irreversible “jailed” state with a demonstrated risk of insulation damage. TTVIV presents the highest risk of mechanical lead failure due to the metal-on-metal interface.

Strategy	Primary Mechanism	Lead Jailing Risk	Lead Interaction Type	New PPM Rate	Lead Extraction Feasibility Post-Proc	Key Lead-Related Concern
T-TEER	Leaflet Approximation	Low	“Innocent Bystander” or Displaced	<1–3%	Preserved	Grasping/Entanglement (Rare)
TTVR	Orthotopic Replacement	High (Obligate)	Metal-on-Tissue Compression	~27.8%	Precluded	Insulation Breach (13%); High PPM rate; No future access
Annuloplasty	Annular Reduction	Moderate	Impingement/Anchor collision	Low	Difficult but Possible	Anchor penetration of lead; Septal crowding
CAVI	Heterotopic Valve	High (SVC)	Stent Jailing (SVC segment)	Low	Precluded (High Risk)	Loss of venous access; SVC entrapment
TTVIV	Valve-in-Valve	Obligate	Metal-on-Metal Compression	Low	Precluded	High rate of lead fracture (10.7%); Shearing forces

Abbreviations: CAVI: caval valve implantation; PPM: permanent pacemaker; SVC: superior vena cava; T-TEER: transcatheter tricuspid edge-to-edge repair; TTVIV: transcatheter tricuspid valve-in-valve (implantation); TTVR: transcatheter tricuspid valve replacement.

**Table 3:** Risk factors for cardiac implantable electronic device (CIED) infection can be categorized into three main domains: patient-related, device-related, and procedure-related.

Risk Factors for CIED Infections		
Patient-Related	Device-Related	Procedure-Related
End-stage renal disease Prior CIED infection Fever prior to implant Corticosteroid or other immunosuppressive therapy Chronic obstructive pulmonary disease Malignancy Diabetes	Multiple transvenous leads Repeated prior procedures Device type (CRT > ICD > pacemaker) Abdominal pocket	Prolonged procedure duration Anticoagulation use Non-electrophysiology laboratory setting

Abbreviations: CIED: cardiac implantable electronic device; ICD: implantable cardioverter-defibrillator; CRT: cardiac resynchronization therapy.

**Table 4:** Comparison of Guideline Recommendations for CIED Management in Patients with Tricuspid Valve Disease.

Clinical Domain	2025 EHRA/EAPCI Statement	2025 AUC/2022 Leadless Position Paper	2021 ESC Pacing Guidelines	2020 ACC/AHA VHD Guidelines	2017 HRS Lead Extraction Consensus
TR and CIED interaction	Central focus; distinguishes CIED-induced vs. CIED-incidental and mixed TR; defines “red flags” for lead jailing	Not the primary focus	Recognized as a complication; echocardiography-centered work-up	Acknowledged but limited mechanistic detail	Indirectly addressed (extraction may improve TR)
Heart Team/Multidisciplinary approach	Mandated for all TTVI candidates	Endorsed for complex candidacy decisions	Recommended for complex scenarios	Required for valvular intervention	Emphasized for high-risk extraction; institutional preparedness required
Lead management strategy	TLE viable even in elderly; risk stratification framework provided	Extraction risk favors a leadless/extravascular approach	Epicardial leads at TV surgery (Class IIa, Level C); CS pacing for mechanical TV prostheses	Not addressed	Core document: procedural endpoints, safety standards, program infrastructure
Alternative pacing/ICD systems	Leadless pacing, CS pacing, epicardial leads, S-ICD/EV-ICD endorsed	Leadless: AUC score 6 post-TV surgery; candidacy framework for leadless/S-ICD/EV-ICD	CS and epicardial strategies acknowledged	Not addressed	Not the primary focus
Monitoring and follow-up	Frequent follow-up essential for jailed leads, especially PM-dependent/ICD patients	Standard device follow-up	Remote monitoring recommended (Class I, Level A)	Not addressed	Post-extraction surveillance addressed

Abbreviations: AUC = Appropriate Use Criteria; CS = coronary sinus; EV-ICD = extravascular ICD; PM = pacemaker; S-ICD = subcutaneous ICD; TLE = transvenous lead extraction; TR = tricuspid regurgitation; TV = tricuspid valve; TTVI = transcatheter tricuspid valve intervention; VHD = valvular heart disease.

**Table 5:** Future directions in the management of patients with CIEDs undergoing TTVI.

**Research Priorities**			
**Domain**	**Current Limitation**	**Future Direction**	**Potential Impact**
Evidence generation	No RCTs comparing extraction vs. lead preservation; limited long-term jailed-lead data	Multicenter randomized trials or prospective registries with prespecified treatment algorithms and standardized endpoints	Guideline-level recommendations for extract-vs-jail decisions
Terminology standardization	Inconsistent definitions of CIED-induced vs. CIED-incidental TR across studies	Consensus terminology distinguishing direct mechanical interference from functional mechanisms	Improved cross-study comparability and treatment effect estimation
Predictive modeling	No validated tools to identify patients benefiting from extraction or stratify procedural risk	AI/ML algorithms integrating clinical, imaging, and device variables for individualized decision support	Personalized extract-vs-jail recommendations with quantified uncertainty
Collaborative infrastructure	Fragmented data collection; variable imaging protocols; short follow-up	International registries with harmonized protocols, dedicated jailed-lead cohorts, and ≥2-year follow-up	Robust evidence base for guideline development and quality benchmarking
**Technological Innovations**			
**Domain**	**Current Limitation**	**Future Direction**	**Potential Impact**
Implantation strategies	Lead placement without systematic consideration of TV interaction	Real-time 3D echo/ICE-guided implantation to avoid leaflet-interfering trajectories	Primary prevention of CIED-induced TR
Leadless and extravascular platforms	Limited physiologic pacing options without transvalvular hardware	Modular leadless PM + S-ICD/EV-ICD systems; leadless CSP; fully leadless CRT	Complete valve-sparing device therapy across pacing and defibrillation indications
TTVI device design	Current devices are not engineered for lead coexistence	Lead-accommodating features: commissural alignment strategies, dedicated lead channels	Safe jailing with preserved lead function and valve durability
Smart lead technology	Lead–valve interaction detected only after clinical TR develops	Sensor-equipped leads detecting early mechanical stress, impedance changes, or micro-motion	Preemptive intervention before symptomatic TR
Computational planning	Empiric decision-making without patient-specific simulation	Digital twin modeling to simulate extraction risk, jailing outcomes, and device–anatomy interaction	Virtual procedural planning and complication prediction
Regenerative and gene therapy	No biologic approaches to CIED-induced valve injury	Tissue-engineered leaflet repair; gene therapy targeting fibrosis; biocompatible lead coatings	Restoration of native valve function; prevention of lead-related fibrosis

Abbreviations: EV-ICD = extravascular ICD; ICE = intracardiac echocardiography; RCT = randomized controlled trial; S-ICD = subcutaneous ICD; TR = tricuspid regurgitation; TTVI = transcatheter tricuspid valve intervention.

## Data Availability

No new data were created or analyzed in this study. Data sharing is not applicable to this article.

## Abbreviations

3D	Three-dimensional
ACC/AHA/ASE/HFSA/HRS/SCAI/SCCT/SCMR	American College of Cardiology/American Heart Association/American Society of Echocardiography/Heart Failure Society of America/Heart Rhythm Society/Society for Cardiovascular Angiography and Interventions/Society of Cardiovascular Computed Tomography/Society for Cardiovascular Magnetic Resonance
AF	Atrial fibrillation
AI	Artificial intelligence
ATP	Antitachycardia pacing
AV	Atrioventricular
CAVI	Caval valve implantation (heterotopic caval valve implantation)
CE	Conformité Européenne (CE marking)
CIED	Cardiac implantable electronic device
CRT	Cardiac resynchronization therapy
CRT-D	CRT with defibrillator
CS	Coronary sinus
CT	Computed tomography
ECAVI/EHRA	European Association of Cardiovascular Imaging/European Heart Rhythm Association (commonly abbreviated EACVI/EHRA; appears as ECAVI/EHRA in your manuscript)
EHRA	European Heart Rhythm Association
EHRA/EAPCI	European Heart Rhythm Association/European Association of Percutaneous Cardiovascular Interventions
EHRA/HRS/LAHRS/APHRS	European Heart Rhythm Association/Heart Rhythm Society/Latin American Heart Rhythm Society/Asia Pacific Heart Rhythm Society
ESC	European Society of Cardiology
EU	European Union
EV-ICD	Extravascular ICD
FDA	U.S. Food and Drug Administration
HRS	Heart Rhythm Society
ICD	Implantable cardioverter-defibrillator
ICE	Intracardiac echocardiography
IVC	Inferior vena cava
LV	Left ventricle
PH	Pulmonary hypertension
PM	Pacemaker
RA	Right atrium
RCT	Randomized controlled trial
RV	Right ventricle
S-ICD	Subcutaneous ICD
SVC	Superior vena cava
T-TEER	Transcatheter tricuspid edge-to-edge repair
TAVR	Transcatheter aortic valve replacement
TEE	Transesophageal echocardiography
TEER	Transcatheter edge-to-edge repair
TLE	Transvenous lead extraction
TR	Tricuspid regurgitation
TRILUMINATE	TRILUMINATE trial
TRISCEND	TRISCEND trial
TRIUMINATE	TRIUMINATE study/trial
TTE	Transthoracic echocardiography
TTVI	Transcatheter tricuspid valve intervention
T-VIV	Tricuspid valve-in-valve
TTVR	Transcatheter tricuspid valve replacement
TV	Tricuspid valve
VHD	Valvular heart disease
VIVID	Valve-in-Valve International Data (VIVID) registry
